# Temporal trends of hemoglobin among pregnant women: The Mutaba’ah study

**DOI:** 10.1371/journal.pone.0295549

**Published:** 2023-12-08

**Authors:** Aminu S. Abdullahi, Abubaker Suliman, Moien AB Khan, Howaida Khair, Saad Ghazal-Aswad, Iffat Elbarazi, Fatima Al-Maskari, Tom Loney, Rami H. Al-Rifai, Luai A. Ahmed

**Affiliations:** 1 Institute of Public Health, College of Medicine and Health Sciences, United Arab Emirates University, Al Ain, United Arab Emirates; 2 Department of Family Medicine, College of Medicine and Health Sciences, United Arab Emirates University, Al Ain, United Arab Emirates; 3 Department of Obstetrics & Gynecology, College of Medicine and Health Sciences, United Arab Emirates University, Al Ain, United Arab Emirates; 4 Obstetrics and Gynecology Department, Tawam Hospital, Al Ain, United Arab Emirates; 5 Zayed Centre for Health Sciences, United Arab Emirates University, Al Ain, United Arab Emirates; 6 College of Medicine, Mohammed Bin Rashid University of Medicine and Health Sciences, Dubai, United Arab Emirates; University of the Witwatersrand, SOUTH AFRICA

## Abstract

**Background:**

Low hemoglobin (Hb) level is a leading cause of many adverse pregnancy outcomes. Patterns of changes in Hb levels during pregnancy are not well understood.

**Aim:**

This study estimated Hb levels, described its changing patterns across gestational trimesters, and identified factors associated with these changes among pregnant women.

**Materials and methods:**

Data from the ongoing maternal and child health cohort study–The Mutaba’ah Study, was used (N = 1,120). KML machine learning algorithm was applied to identify three distinct cluster trajectories of Hb levels between the first and the third trimesters. Descriptive statistics were used to profile the study participants. Multinomial multivariable logistic regression was employed to identify factors associated with change patterns in Hb levels.

**Results:**

The three identified clusters–A, B and C–had, respectively, median Hb levels (g/L) of 123, 118, and 104 in the first trimester and 119, 100, and 108 in the third trimester. Cluster ’A’ maintained average normal Hb levels in both trimesters. Cluster ’B’, on average, experienced a decrease in Hb levels below the normal range during the third trimester. Cluster ’C’ showed increased Hb levels in the third trimester but remained, on average, below the normal range in both trimesters. Pregnant women with higher gravida, diabetes mellitus (type 1 or 2), nulliparity or lower level of education were more likely to be in cluster ’B’ than the normal cluster ’A’. Pregnant women who reported using iron supplements before pregnancy or those with low levels of education. were more likely to be in cluster ’C’ than the normal cluster ’A’.

**Conclusion:**

The majority of pregnant women experienced low Hb levels during pregnancy. Changes in Hb levels during pregnancy were associated with parity, gravida, use of iron before pregnancy, and the presence of diabetes mellitus (type 1 or 2).

## Introduction

Hemoglobin (Hb) is an iron-containing protein that plays an essential role in the biological system as a two-way respiratory transporter [1,2]. Although the Hb levels remain relatively stable during adulthood, they are influenced by demographic factors (e.g., age, sex, and race), lifestyle factors (e.g., smoking and diet), physiological changes such as pregnancy, and the additional need for iron for placental and fetal growth [3,4]. Pregnancy comes with various events and changes, one of which is a ~50% increase in plasma volume [3]. This considerable increase in plasma volume does not involve an equivalent increase in the number of red blood cells leading to decreased concentration of the red blood cells and subsequently in the Hb levels [3]. This decrease in Hb levels during pregnancy may lead to anemia, defined by a Hb level lower than 110 g/L [5].

In 2019, global anemia prevalence was 29.9% (95% confidence interval (CI) 27.0%, 32.8%) in women of reproductive age, equivalent to over half a billion women aged 15–49 years. The prevalence was 29.6% (95% CI: 26.6%, 32.5%) in non-pregnant women of reproductive age and 36.5% (95% CI: 34.0%, 39.1%) in pregnant women [6]. The anemia prevalence among pregnant women in the Gulf countries (Bahrain, Kuwait, Oman, Qatar, Saudi Arabia and the United Arab Emirates) between 1978 and 1997 ranged from 23% to 54% [7]. The economic and healthcare costs associated with anemia can also be high in countries burdened with a high prevalence of anemia [8].

Low Hb levels and anemia are linked with maternal and neonatal morbidity and mortality [1,2,9]. Increased risks of adverse pregnancy outcomes, including preterm delivery, low birth weight, cesarean delivery, preeclampsia, antenatal admissions, and longer duration of hospitalization, were reported to be driven by anemia during pregnancy [9–12]. The risk of some adverse maternal and neonatal effects, such as low birth weight and preterm birth, increases proportionately with increasing severity of anemia, being more common in mothers with severe anemia (Hb < 70 g/L) [13]. Various factors have been reported to be associated with anemia in pregnancy, including sub-optimal birth spacing, lack of antenatal care, gestational age, and not taking iron-folate supplements [14,15].

A clear understanding of changes in Hb level during pregnancy is of great clinical and public health importance. It will improve the prevention and management of consequent conditions such as iron deficiency anemia (IDA). Most anemia estimates for the Gulf countries are 25–40 years old [7], and all the Gulf countries have experienced considerable sociodemographic and economic changes since then. Furthermore, previous (more recent) studies had relatively small sample sizes and were cross-sectional and did not provide any insight into the temporal changes in anemia or Hb level, especially during pregnancy [16–18]. In a cohort of pregnant women, the aim of this study was to estimate the Hb level, characterize changes across gestational periods, and identify factors associated with Hb level changes during pregnancy.

## Methods

### Study design, setting and participants

This analysis is based on data from the Mutaba’ah Study, the largest ongoing prospective cohort study of mother and child health in the United Arab Emirates (UAE). A detailed description of the Mutaba’ah Study can be found elsewhere [19]. In brief, the study recruits and follows pregnant women and their newborns until the child reaches 18 years. All pregnant women from the Emirati population aged 18 years and above, residents of Al Ain city, and able to provide informed consent and their newborns are eligible to participate in the study. Recruitment started in May 2017 and is taking place in the two major hospitals in the city. Participants in the Mutaba’ah Study participated purely voluntarily and received complete information about the study. All participants provided written informed consent. The study was approved by the United Arab Emirates University Human Research Ethics Committee (ERH-2017-5512) and the Abu Dhabi Health Research and Technology Ethics Committee (DOH/CVDC/2022/72).

### Variables and measurements

This analysis included all pregnant women who were recruited and gave birth between May 2017 and May 2022. Participants were followed up during pregnancy, and data were collected using a self-administered questionnaire completed at the first contact with the pregnant woman and medical records extraction performed immediately after the woman gave birth. Questionnaire data used in this analysis covered information on socio-demographics, psycho-socials, pregnancy behaviors, and previous pregnancies. The pregnant women were classified as having higher than secondary education if they selected "Vocational/Diploma", "Bachelors", "Masters" or "Doctorate", and secondary or lower education if selected "Illiterate", "Never attended school", "Primary" or "Secondary" in the question about education level. Employment status of the participants was coded as employed ("Employed", "Self-employed") or unemployed ("Student", "Housewife", "Retired", "Seeking employment"). Statuses of having a planned pregnancy, infertility treatment and worry regarding birth were coded as yes or no. Self-reported daily folate and iron use, before and during this pregnancy, was coded as yes if the pregnant women indicated “Daily” use of the supplements and no if the response was “Weekly”, “Monthly” or “Never used”.

Hb concentration, body mass index, parity, and gravidity were extracted from medical records. Gravida refers to the number of conceptions a woman has had including the current pregnancy, while parity refers to the total number of births. Routine Hb measurements taken during the first and third trimesters were considered in this analysis. In this study, anemia was defined as a Hb level lower than 110 g/L for pregnant women [5].

### Identification of Hb longitudinal trajectories

KML algorithm [20], an implementation of the unsupervised machine learning algorithm K-means, was applied to identify distinct cluster trajectories of Hb levels during pregnancy. The KML method is a non-parametric technique specifically designed to cluster longitudinal data, which does not require certain assumptions regarding the trajectories’ shape. It has been effectively employed in other studies to correctly identify distinct clusters of various metabolic and anthropometric measures in cohorts of study subjects [21,22]. In brief, KML executes K-means for a different number of clusters, by default two to six clusters, permitting multiple draws for each cluster and varying the initialization methods (i.e., starting conditions) in each run for each number of clusters. The quality of the identified clusters was then assessed using different non-parametric criteria. This study chose the optimal number of clusters based on the Calinski–Harabasz criterion [23]. The pregnant women were clustered into three (A, B and C) cluster trajectories based on their Hb levels in the first and third trimesters.

### Statistical analysis

Descriptive statistics were used to profile the study participants. Frequencies and percentages were used for categorical variables. For the continuous variables, median and interquartile ranges were used because their distribution was skewed as tested by Kolmogorov–Smirnov test. The three identified clusters of women were further compared across demographic and maternal variables using the Chi-squared test and Kruskal-Wallis ANOVA for categorical and continuous variables, respectively. Multivariable multinomial logistic regression was used to identify factors associated with being in cluster B or C compared to cluster A. Adjusted odds ratios (aOR) with 95% confidence intervals were reported. All statistical tests were two-sided; p-values <0.05 were considered statistically significant. All analyses were performed using R software version 4.0.3 and KML package version 2.4.1 [24].

## Results

A total of 1,120 pregnant women with available Hb measurements in the first and third trimesters were included in the analysis. The median age of the women was 30.8 years (IQR = 25.9–35.7). Overall, one-quarter (25.1%) of pregnant women were anemic (Hb <110 g/L) during the first trimester. This estimate doubled to half (51.1%) of pregnant women during the third trimester. Moreover, nearly two-thirds (64.8%) of pregnant women who were anemic during the first trimester remained anemic during the third trimester. Of the women who were non-anemic in the first trimester, nearly half (47%) developed anemia during the third trimester.

### Trajectory clusters and the changes in Hb level across gestational trimesters

**Fig 1:**
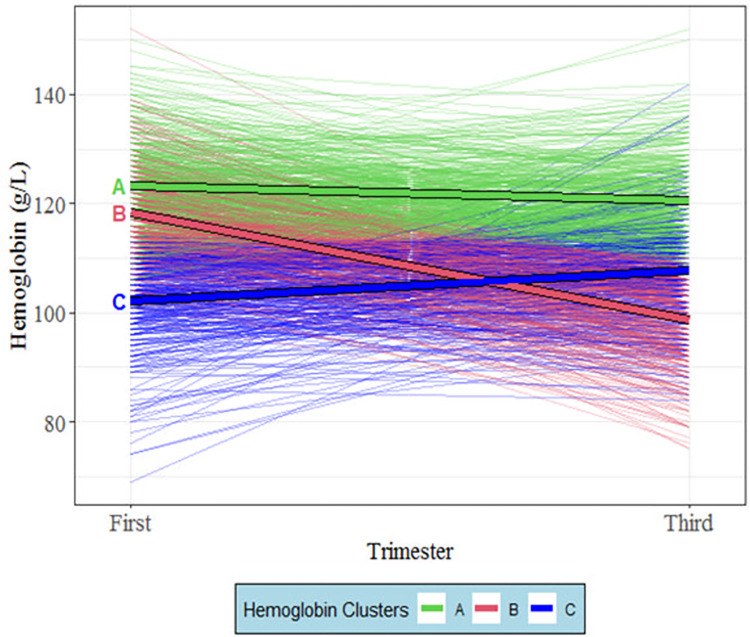
Three clusters of hemoglobin level trajectories between the first and third trimesters in pregnant women. The Mutaba’ah Study. The solid line represents the cluster’s mean Hemoglobin level.

**Fig 2:**
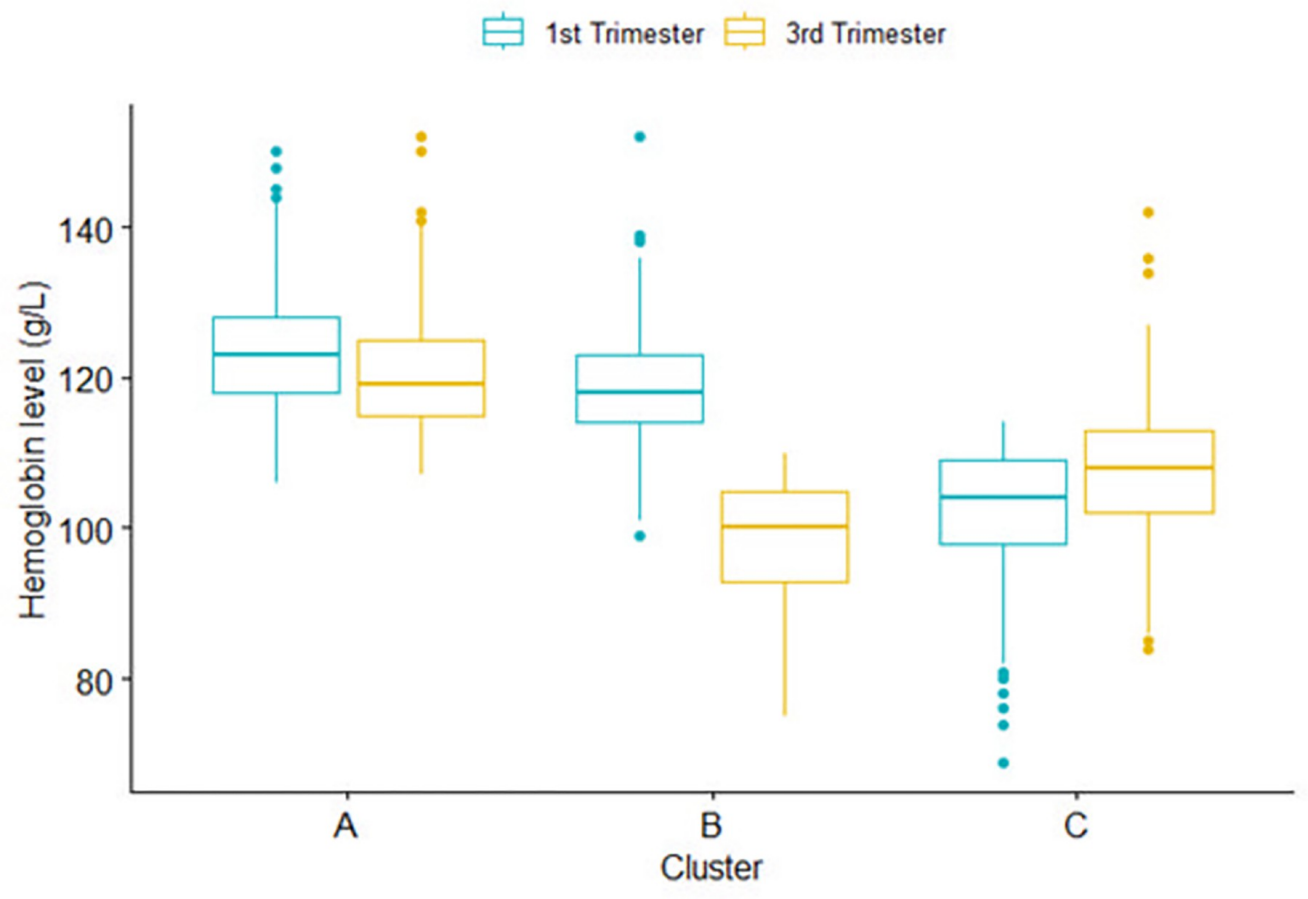
Median and interquartile range of hemoglobin level across the three clusters of hemoglobin level trajectories between the first and third trimesters in pregnant women. The Mutaba’ah Study.

Changes in Hb levels between the first and third trimesters and the identification of the clusters are displayed in Fig 1. The median Hb levels (g/L) in the first trimester, across the three cluster trajectories (A, B and C), were 123 [IQR = 118–128], 118 [IQR = 114–123], and 104 [IQR = 98–109], respectively (Fig 2). In the third trimester, these values were 119 [IQR = 115–125], 100 [IQR = 93–105], and 108 [IQR = 102–113]. Despite the slight decrease, pregnant women in cluster A maintained average normal Hb levels in both the first and the third trimesters. Those in cluster B, on average, experienced a decrease in their Hb level below the normal range during the third trimester. Cluster C showed increased Hb levels in the third trimester but remained, on average, below the normal range in both trimesters. Hence, clusters A, B, and C will often be referred to as *normal* cluster ’A’, *decreased* cluster ’B’ and *increased* cluster ’C’, respectively, in this paper.

### Characteristics of different trajectory clusters

The demographic and clinical characteristics of the pregnant women considered in this study were summarized and presented in Table 1 by cluster trajectories. Nearly two-thirds (63.8%) of the pregnant women had more than two pregnancies, with statistically significant differences across the clusters (P = 0.01). Approximately half (49.2%) of pregnant women reported having at least a tertiary-level education. Those in the normal cluster ’A’ were significantly more educated, comprising 41% of those with a tertiary-level education (P = 0.021) and more women (22%) used iron supplements daily during pregnancy than before (14.8%). While there were no statistically significant differences across the clusters regarding daily iron supplementation during pregnancy, the use of daily iron supplements before the current pregnancy was significantly different (P = 0.040), with the highest proportion among those in the *increased* cluster ’C’ (34.5%). There were no statistically significant differences across the women clusters in terms of age, previous infertility treatment, diabetes mellitus (type 1 or 2), daily use of folic acid before pregnancy, and the daily use of iron during pregnancy.

**Table 1:** Demographic and clinical characteristics of 1,120 pregnant women by the three identified clusters of hemoglobin level trajectories between the first and third trimesters. The Mutaba’ah Study.

	Total	Hemoglobin level trajectory clusters			*p- value*
		A *(Normal & stable Hb level)*	B *(Decreased Hb level)*	C *(Increased Hb level)*	
**N**	1,120	418 (37.3%)	405 (36.2%)	297 (26.5%)	
**Age,** median (IQR)	30.8 (25.9–35.7)	30.8 (26.1–35.0)	30.1 (25.6–35.7)	31.9 (26.2–37.2)	*0*.*127*
**BMI,** median (IQR)	27.5 (23.9–31.5)	27.4 (24.1–31.1)	27.9 (24.3–32.1)	26.7 (23.2–31.2)	*0*.*089*
**Gravida**					
≤2	405 (36.2%)	170 (42.0%)	147 (36.3%)	88 (21.7%)	***0*.*01***
>2	715 (63.8%)	248 (34.7%)	258 (36.1%)	209 (29.2%)	
**Parity**					
0	284 (25.4%)	112 (39.4%)	112 (39.4%)	60 (21.1%)	*0*.*056*
≥1	836 (74.6%)	306 (36.6%)	293 (35.0%)	237 (28.3%)	
**Education**					
Secondary or lower	519 (50.8%)	171 (32.9%)	200 (38.5%)	148 (28.5%)	***0*.*021***
Higher than secondary	503 (49.2%)	208 (41.4%)	169 (33.6%)	126 (25.0%)	
**Employment status**					
Unemployed	681 (66.6%)	245 (36.0%)	248 (36.4%)	188 (27.6%)	*0*.*61*
Employed	341 (33.4%)	133 (39.0%)	121 (35.5%)	87 (25.5%)	
**Planned pregnancy**					
No	469 (45.1%)	166 (35.4%)	182 (38.8%)	121 (25.8%)	*0*.*315*
Yes	571 (54.9%)	221 (38.7%)	196 (34.3%)	154 (27.0%)	
**Worry about giving birth**					
No	333 (32.9%)	109 (32.7%)	123 (36.9%)	101 (30.3%)	*0*.*108*
Yes	679 (67.1%)	264 (38.9%)	243 (35.8%)	172 (25.3%)	
**Infertility treatment**					
No	925 (90.2%)	347 (37.5%)	336 (36.3%)	242 (26.2%)	*0*.*552*
Yes	100 (9.8%)	32 (32.0%)	40 (40.0%)	28 (28.0%)	
**Diabetes mellitus** (type 1 or 2)					
No	989 (95.5%)	375 (97.2%)	351 (93.6%)	263 (95.6%)	*0*.*062*
Yes	47 (4.5%)	11 (2.8%)	24 (6.4%)	12 (4.4%)	
**Daily folate before pregnancy**					
No	612 (78.8%)	229 (37.4%)	226 (36.9%)	157 (25.7%)	*0*.*827*
Yes	165 (21.2%)	66 (40.0%)	59 (35.8%)	40 (24.2%)	
**Daily iron before pregnancy**					
No	675 (88.9%)	675 (88.9%)	675 (88.9%)	675 (88.9%)	***0*.*040***
Yes	675 (88.9%)	675 (88.9%)	675 (88.9%)	675 (88.9%)	
**Daily folate during pregnancy**					
No	519 (67.3%)	185 (35.6%)	197 (38.0%)	137 (26.4%)	*0*.*452*
Yes	252 (32.7%)	101 (40.1%)	92 (36.5%)	59 (23.4%)	
**Daily iron during pregnancy**					
No	606 (80.8%)	227 (37.5%)	227 (37.5%)	152 (25.1%)	*0*.*900*
Yes	144 (19.2%)	51 (35.4%)	56 (38.9%)	37 (25.7%)	

Note: Frequencies in some variables may not add to 1,120 due to missing data.

### Factors associated with being in the decreased cluster ’B’

Multivariable multinomial logistic regression models were used to identify factors associated with negative or positive change in Hb (Table 2). The *normal* cluster ’A’ was used as the reference cluster, which maintained relatively stable and normal Hb levels during both trimesters. Pregnant women with a gravida of above two were nearly twice as likely to be in the *decreased* cluster ’B’ than in the normal cluster ’A’ (adjusted odds ratio [aOR]: 1.88, 95% CI: 1.07–3.31) compared to those with a gravida of ≤2. Parous women were about 50% less likely to be classified in the *decreased* cluster ’B’ than in the normal cluster ’A’ (aOR: 0.49, 95% CI: 0.28–0.88) compared to the nulliparous. Compared to non-diabetic women, pregnant women with diabetes mellitus (type 1 or 2) were more than three times more likely to be classified in the *decreased* cluster ’B’ than the *normal* cluster ’A’ (aOR: 3.46, 95% CI: 1.22–9.84). Women with a level of education above high school were less likely to be in the *decreased* cluster ’B’ compared to those with a lower level of education (aOR: 0.55, 95% CI: 0.37–0.80). Age, body mass index, and the use of iron before pregnancy were not significantly associated with being in the decreased cluster ’B’ than the *normal* cluster ’A’ (P ≥ 0.05) (Table 2).

**Table 2:** Multivariable multinomial logistic regression for factors associated with being in hemoglobin level trajectory clusters B or C compared to cluster A. The Mutaba’ah Study.

	B *versus* A	C *versus* A
**Age**	0.99 [0.96, 1.03]	1.01 [0.98, 1.05]
**Body mass index**	1.01 [0.98, 1.05]	0.97 [0.94, 1.01]
**Gravida**		
≤2	1	1
>2	**1.88 [1.07, 3.31]** ^*^	1.64 [0.89, 3.02]
**Parity**		
0	1	1
≥1	**0.49 [0.28, 0.88]** ^*^	0.99 [0.52, 1.89]
**Diabetes mellitus** (type 1 or 2)		
No	1	1
Yes	**3.46 [1.22, 9.84]** ^*^	1.84 [0.55, 6.13]
**Iron before pregnancy**		
No	1	1
Yes	1.38 [0.69, 2.78]	**2.07 [1.01, 4.24]** ^*^
**Education**		
Secondary or lower	1	1
Higher than secondary	**0.55 [0.37, 0.80]** ^**^	**0.59 [0.39, 0.89]** ^*^

Hemoglobin (Hb) level trajectories between the first and third trimesters: cluster A (Normal & stable Hb level), cluster B (Decreased Hb level), cluster C (Increased Hb level).

^*^ p < 0.05

^**^ p < 0.01

The models were additionally adjusted for daily iron use during pregnancy.

### Factors associated with being in the increased cluster ’C’

The use of iron before pregnancy and the education level of the pregnant women were significantly associated with being in the *increased* cluster ’C’ as opposed to the *normal* cluster ’A’. Women who daily used iron supplements before the current pregnancy were about twice as likely to be classified in the *increased* cluster ’C’ than the *normal* cluster ’A’ compared to those who did not (aOR: 2.07, 95% CI: 1.01–4.24). Those with an education level higher than secondary school were about 40% less likely to be in the *increased* cluster ’C’ rather than the *normal* cluster ’A’ compared to those with a lower level of education (aOR: 0.59, 95% CI: 0.39–0.89). Age, body mass index, gravida, parity, and diabetes mellitus (type 1 or 2) status were not associated with being in the *increased* cluster ’C’ than the *normal* cluster ’A’ (P>0.05) (Table 2).

## Discussion

In this study, we examined pregnant women’s Hb levels during the first and third gestational trimesters, identified three clusters based on their Hb trajectory during the pregnancy, and explored the factors associated with being in these clusters.

In this cohort of pregnant women, one in every five experienced anemia during the first trimester, while in the third trimester, one in every two developed anemia. Approximately half of the pregnant women who were not anemic in the first trimester became anemic during the third trimester, and two-thirds (68%) of those who were anemic during the first trimester remained anemic during the third trimester. The present findings concur with previous studies in Gulf countries, indicating a higher prevalence of anemia during the third trimester compared to the first trimester [17,18]. Specifically, a cross-sectional study involving 390 pregnant women in Saudi Arabia estimated the prevalence of anemia among pregnant women at 34.1%. The prevalence was higher (40.3%) among those in the third trimester compared to those in the first trimester (32.3%) [17]. Another study from Oman reported the prevalence of anemia among 296 pregnant women to be about 42%, with a higher prevalence among those in the third trimester (27%) compared to those in their first trimester of pregnancy (9%) [18]. The levels of heterozygotes and homozygotes, the alleles of the two common variants of the hemochromatosis gene that enhance iron absorption, are lower in the Gulf countries than in the European populations. This could explain, in part, the high frequency of IDA within this region. Moreover, there is a high prevalence of alpha- and beta-thalassemia traits in healthy women (20–30% and 16%, respectively) compared to approximately 5% of the global population [25]. In addition, late initiation of antenatal care reported in this population [26] might contribute to the higher rate of anemia during the third trimester. Moreover, a higher amount of iron is required during the last trimester (7.5 mg/day) than the first trimester (0.8 mg/day), thereby making it easier to treat iron deficiency anemia during the first trimester as compared to the later trimesters [27–29].

Although it is common for pregnant women to have slightly low hemoglobin levels because of the increased blood volume, the study’s findings indicate that many women who were anemic during the first trimester remained anemic during the third trimester. It could be attributed to the failure rate of anemia treatment, as reported by numerous studies [30,31], although it is difficult to categorize the reasons for this treatment failure rate. It has been hypothesized, however, that this could be related to several factors, including treatment compliance issues, micronutrient deficiencies, and co-morbid conditions that could negatively impact absorption [32,33].

This study investigated factors associated with changes in Hb levels between the first and third trimesters. Having normal Hb levels in the first trimester, women with a higher gravida were more likely to become anemic in the third trimester. Although many [34,35] but not all [36] studies indicated that parity increases the likelihood of anemia, our findings showed that nulliparous women were more likely to experience a decrease in Hb levels below the normal range in the third trimester, than their parous counterparts. Moreover, pregnant women with a lower education level or diabetes mellitus (type 1 or 2) were three times more likely to experience a decrease in Hb beyond the normal level in the third trimester, a finding similar to other studies [37,38].

Overall, the anemic trend in cluster B warrants the early identification of those at risk and interference to prevent the deterioration of the Hb levels from the first to the third trimester. To improve perinatal outcomes and guide practice, policy, and research, anemia during pregnancy should be targeted at different levels. The social-ecological model as a multidimensional framework is highly congruent with many intervention-based disease prevention programs [39]. Various interventions should be implemented at the individual, community, societal, and stakeholder levels. Anemia during pregnancy can be reduced by implementing interventions to spread information and advocacy, and counseling for energy-balanced, nutrient-dense diets and supplements. There is a need for more campaigns to address anemia during pregnancy and the importance of nutritional education to pregnant women to maintain a healthy diet rich in iron to prevent anemia. To improve the diet of pregnant women, mobile health interventions can be utilized as a means of providing reminders and educational tips. It is also important to follow up with pregnant women who are at a high risk of anemia as well as to provide iron supplements to pregnant women at risk of anemia. Targeted preconception and prenatal counseling are needed to reduce the burden of anemia during pregnancy [40]. Premarital counseling and preconception care should also be improved to address the primordial prevention of anemia during pregnancy. Pregnant women can also benefit from community-based interventions such as peer groups or community health workers who provide support and motivation to improve their overall health. Furthermore, guidelines should include prevention strategies based on policy and interventions from stakeholders.

To our knowledge, this is the first study to examine the changing trends in hemoglobin concentration across gestations among women from the Emirati population. Furthermore, the study identified the incidence of anemia among a large representative sample of pregnant women at different gestational ages. Moreover, to our knowledge, this is the first study in the United Arab Emirates to categorize hemoglobin concentration in pregnant women during their first and third trimesters using a machine learning algorithm. Clinical practice can benefit from the results using machine learning. Nonetheless, the study has few limitations. The lifestyle and sociodemographic factors were self-reported, so recall or social desirability bias may exist. The analysis did not investigate the type of anemia or the pregnancy and fetal outcomes in women with anemia during pregnancy. However, the prospective design of the Mutaba’ah Study will allow for gauging maternal and fetal outcomes associated with anemia during pregnancy in future analyses.

## Conclusion

In summary, the study revealed that more than one-quarter of pregnant women were anemic during the first trimester, half were anemic during the third trimester, and more than two-thirds of women who were anemic during the first trimester were anemic during the third trimester. Changes in Hb levels during pregnancy were associated with parity, gravida, use of iron before pregnancy, and having diabetes mellitus (type 1 or 2). Anemia remains a significant challenge, requiring high–quality surveillance and interventions. Policies and strategies should encourage pregnant women to eat a nutritionally balanced diet before and during pregnancy to optimize outcomes for both the mother and the child.

## Supporting information

S1 Checklist STROBE statement—Checklist of items that should be included in reports of observational studies. (DOCX)
